# Perceived temporal reorganization during psychiatric hospitalization among people with schizophrenia: a qualitative interview study

**DOI:** 10.3389/fpsyt.2026.1869125

**Published:** 2026-08-18

**Authors:** Yujuan Qin, Qin Mao, Hualei Li, Faqiang Zhang, Suhong Wang, Dongyang Wang, Jianying Wang

**Affiliations:** 1 School of Nursing, Henan Medical University, Xinxiang, China; 2 The Second Affiliated Hospital of Henan Medical University, Xinxiang, China; 3 Department of Nursing, The Third People’s Hospital of Henan Province, Zhengzhou, China

**Keywords:** psychiatric hospitalization, psychosocial recovery, qualitative study, schizophrenia, time perspective

## Abstract

**Background:**

Schizophrenia involves not only disturbances in thought, perception, and self-experience, but also disruptions in subjective temporality. During psychiatric hospitalization, recovery experiences may involve not only symptom stabilization but also patients’ efforts to make sense of continuity between past, present, and future. This study explored how inpatients with schizophrenia described their time perspective during hospitalization.

**Methods:**

This qualitative interview study recruited 15 clinically stable inpatients diagnosed with schizophrenia from four closed wards of a mental health center in China. Semi-structured interviews were conducted and analyzed using reflexive thematic analysis informed by the concept of time perspective.

**Results:**

Five themes were generated: (1) the duality of the past perspective—emotional refuge versus traumatic intrusion; (2) the reshaping of present experience—from perceived stagnation to relational awakening; (3) the folding and extension of future images—contraction under uncertainty and gradual reconstruction; (4) coping with temporal distress—passive endurance and active self-soothing; and (5) perceived shifts in temporal organization—emerging agency and changing self-understanding. Participants’ accounts suggested that time perspective during hospitalization was neither uniform nor fixed. Temporal experience was shaped by symptoms, ward restrictions, interpersonal relationships, structured activities, and changing levels of autonomy.

**Conclusions:**

Subjective time perspective in schizophrenia during psychiatric hospitalization appears dynamic and context-sensitive rather than static. Participants’ narratives suggest that perceived temporal reorganization may emerge within the context of hospitalization and is related to how individuals experience symptom changes, meaningful activities, interpersonal support, and everyday agency. These findings highlight the relevance of subjective temporality in understanding patients’ experiences during inpatient psychiatric care.

## 1 Introduction

Schizophrenia (SCZ) is a severe and persistent mental disorder that not only affects an individual’s thinking, perception, and self-awareness, but also profoundly reshapes subjective time experience and the organization of daily life (1, 2). For patients requiring inpatient treatment, recovery involves more than the reduction of positive or negative symptoms; it also involves reorganizing everyday life, restoring a sense of meaning, and re-establishing connection with the world. Recent studies suggest that people with schizophrenia often experience enduring difficulties in the dimension of time. These difficulties are reflected not only in impaired processing of temporal order, duration, and rhythm, but also in disruption of life time, reduced continuity of events, and difficulty integrating experience into a sustainable life trajectory (3). At the same time, the physical space, institutional routines, restrictive measures, reduced autonomy, and interpersonal interactions within inpatient psychiatric settings can substantially shape patients’ hospitalization experiences and subjective recovery processes. Understanding how hospitalized patients with schizophrenia experience and organize time may therefore provide a more complete patient-centered view of recovery (4).

Time perspective refers to the habitual ways in which individuals organize and evaluate experiences in relation to the past, present, and future. In Zimbardo and Boyd’s framework, these orientations include past-positive, past-negative, present-hedonistic, present-fatalistic, and future dimensions. In schizophrenia spectrum disorders, recent quantitative studies suggest that more unbalanced time perspective profiles are associated with greater symptom burden, poorer cognitive performance, and worse daily functioning (5). Specifically, a person’s positive or negative interpretation of past experiences, sense of control or fatalism regarding current life, and the clarity of goals and plans for the future together form a distinctive temporal frame of reference (6). Large-sample studies have shown that people with schizophrenia spectrum disorders tend to deviate from a balanced time perspective, and such imbalance is associated with anxiety or depression, thought disorder, anhedonia, and avolition. Time perspective has also been linked to everyday time use and overall functional level (7). Other studies have found that a more imbalanced time perspective is associated with working memory impairment, whereas a more balanced time perspective may facilitate a better therapeutic alliance (8, 9). Overall, temporal orientations such as a negative past, fatalistic present, and restricted future may provide an important entry point for understanding emotion, behavior, and recovery in people with schizophrenia.

However, important gaps remain in the existing literature on time in schizophrenia. First, most available evidence comes from quantitative studies that focus on associations between time perspective dimensions and symptoms or functioning as measured by standardized scales (10, 11). Although these studies reveal general patterns, they are less able to capture the richness, contradiction, and dynamic change of patients’ time experiences in the context of hospitalization. Second, prior research has mainly focused on the cognitive aspects of time perception or temporal processing, such as interval judgment, whereas far less attention has been paid to how time perspective, a more subjective and meaning-laden construct, is reshaped by illness and hospitalization (12). Most importantly, the inpatient psychiatric ward is a highly structured therapeutic setting, yet the ways in which its daily rhythms, treatment activities, and interpersonal relationships influence patients’ reconstruction of the past, experience of the present, and imagination of the future remain largely unexplored. Existing studies have often adopted a deficit-oriented approach, emphasizing impairments in mental time travel, autobiographical memory specificity, and future episodic imagination among people with schizophrenia, while rarely examining with an open perspective whether time perspective may also shift positively during hospitalization and how such growth might occur (4, 13–16).

Against this background, the present study used a qualitative interview design and reflexive thematic analysis to examine how inpatients with schizophrenia described their past-, present-, and future-oriented experiences during psychiatric hospitalization. Rather than assuming that temporal disturbance in schizophrenia is only a stable deficit, we explored whether participants also described partial temporal reorganization within the structured inpatient environment. In this study, we use the term perceived temporal reorganization to refer to participants’ descriptions of changes in the relationships among past, present, and future experiences during hospitalization. This concept does not indicate complete recovery, symptom improvement, or a balanced time perspective. Rather, it captures how individuals may experience shifts in temporal continuity, meaning, and agency within a particular clinical context.

Specifically, we examined how participants related to the past, experienced the inpatient present, envisioned the near future, coped with difficult or empty time, and made sense of perceived changes in their temporal orientation during hospitalization. By foregrounding patients’ narratives, this study aimed to extend predominantly quantitative work on time perspective in schizophrenia and to generate clinically relevant insights into psychosocial recovery within inpatient psychiatric care.

## 2 Methods

### 2.1 Study design

This study used a qualitative interview design and reflexive thematic analysis informed by time perspective theory. The study aimed to explore changes in time perspective among hospitalized patients with schizophrenia, with particular attention to their past-, present-, and future-oriented experiences, coping strategies, and perceived changes and growth during hospitalization. The study was reported in accordance with the Consolidated Criteria for Reporting Qualitative Research (COREQ).

### 2.2 Setting

The study was conducted from September to November 2025 at the Second Affiliated Hospital of Henan Medical University, Xinxiang, China, a regional mental health medical center and the largest mental health facility in the province. Participants were recruited from four different closed psychiatric wards. As the participants were hospitalized in closed psychiatric wards, their experiences of time were embedded in everyday nursing care, ward routines, interpersonal interactions, and recovery-oriented support. The study was embedded in routine inpatient psychiatric care, and all participants were receiving pharmacological treatment combined with psychosocial rehabilitation.

### 2.3 Participants

Purposive sampling was used to identify information-rich participants. With the assistance of ward psychiatrists, patients diagnosed with schizophrenia and judged to be in a relatively stable phase were invited to participate. The inclusion criteria were: (1) a diagnosis of schizophrenia according to the International Classification of Diseases, 10th Edition (ICD-10) (17); (2) aged 18 to 59 years; (3) clinically stable for at least three months, with a Positive and Negative Syndrome Scale (PANSS) positive subscale score ≤ 21 (18); (4) able to communicate effectively with the researchers and willing to participate voluntarily. The exclusion criteria were: (1) comorbid severe physical illness or alcohol/substance abuse; (2) severe agitation, marked thought disorder, acute psychotic instability, or other conditions that might interfere with meaningful participation.

A total of 15 patients were interviewed. The sample size was determined based on information power rather than a predetermined numerical target (19). Given the focused aim of exploring subjective temporal experiences among hospitalized patients with schizophrenia, the clinical specificity of the sample, the use of time perspective theory as a sensitizing framework, and the depth of semi-structured interviews, each participant provided rich and relevant information. During data collection and analysis, the research team continuously assessed the adequacy of the dataset. After 15 participants had completed the interviews, the dataset was considered sufficient to support the development of the themes presented in this study. The demographic characteristics of the participants are presented in **Table 1**.

**Table 1:** Demographic and clinical characteristics of participants (N = 15).

Characteristic	Value
Age (mean ± SD)	33.5 ± 7.8
Illness duration (years/mean ± SD)	4.7 ± 4.2
Sex (n%)	
Male	8 (53.3%)
Female	7 (46.7%)
Marital status (n%)	
Unmarried	6 (40.0%)
Married	7 (46.7%)
Divorced	2 (13.3%)
Education level (n%)	
Junior high school	4 (26.7%)
High school	2 (13.3%)
Associate degree	7 (46.7%)
Bachelor’s degree	2 (13.3%)

### 2.4 Data collection

Before each interview, the purpose of the study was explained to the participant and written informed consent was obtained. Face-to-face semi-structured interviews were then conducted in a private, quiet room within the ward to minimize disturbance and ensure participant comfort. All interviews were conducted by two experienced researchers. Each interview began with general questions and then moved to more specific exploration based on the participant’s responses. Interviews lasted approximately 30–45 minutes. With participants’ permission, all interviews were audio-recorded. The interviewers also documented details such as tone of voice, pauses, body language, and facial expressions during the interview. Data collection continued until sufficient depth and diversity of experiential accounts had been obtained to address the research aim.

Before recruitment, the responsible psychiatrist confirmed clinical stability and capacity to provide informed consent. Participants were informed that refusal or withdrawal would not affect their treatment, nursing care, or ward privileges. For participants who showed distress or disclosed self-harm-related behavior during the interview, the interviewer paused the interview and informed the responsible clinical team according to the ward safety protocol.

The interviews were conducted by YQ and QM, both nursing researchers with training and experience in qualitative interviewing in mental health settings. Neither interviewer was involved in the participants’ treatment or routine nursing care, and neither had a prior relationship with the participants. Before the interviews, participants were informed that the interviewers were members of the research team and that the study aimed to understand patients’ experiences of time during hospitalization.

The interview guide was developed by the research team based on the study aim and the concept of time perspective. Rather than using a fixed questionnaire format, the interviews were guided by open-ended prompts covering five broad domains: experiences and meanings of the past, perceptions of the present during hospitalization, views of the future, ways of coping with difficult time and emotional distress, and perceived changes and growth across the illness and hospitalization trajectory. Follow-up questions were used flexibly to encourage participants to elaborate on their experiences and to clarify emerging meanings. The detailed interview outline is as follows:

1. “Do you often recall the past? When you think back, what comes to mind more easily?”
2. “When you think about the past (whether good or bad), how does it typically affect your current day, your mood, or your motivation to do things?”
3. “In the ward, what moments make you feel that time is most difficult to endure? And what moments make you feel that time is passing meaningfully?”
4. “What are your current concerns, and what expectations do you have for society, your family, and the nurses to help you?”
5. “How do you view your future, and what plans do you have?”
6. “If you imagine the future, how far after discharge can you picture most clearly? Why this time frame?”
7. “When you are in a bad mood or under stress, what do you do to cope with time? How effective are your most commonly used methods? If they don’t work, what do you do then?”
8. “From the time you fell ill and were hospitalized to the stable phase, how do you feel your relationship with time has changed?”
9. “When did you feel that your experience of time began to change (becoming clearer, more fulfilling, faster, or slower)? What happened that led to this change?”
10. “How are you different now compared to who you were in the past?”

### 2.5 Data analysis

Interviews were transcribed verbatim in Mandarin and checked against the audio recordings for accuracy. Field notes recorded during and immediately after interviews were reviewed alongside transcripts to support contextual understanding. Data were analyzed using reflexive thematic analysis following Braun and Clarke’s six-phase approach (20). The analysis adopted a contextualist orientation, recognizing participants’ accounts as situated experiences shaped by illness, interpersonal relationships, and the inpatient environment. Time perspective theory was used as a sensitizing framework to guide attention to participants’ experiences of the past, present, and future, while allowing themes to be developed inductively from the data.

The research team engaged in repeated familiarization with the transcripts. Initial coding focused on identifying meaningful features of participants’ accounts related to temporal experiences, including how participants described memories, daily routines, future expectations, coping responses, and perceived changes during hospitalization. Codes were not developed as fixed categories for agreement testing; rather, they served as interpretive tools to explore patterns of meaning within the dataset. Candidate themes were developed by examining relationships among codes and considering how they addressed the research question. Themes were repeatedly reviewed against coded extracts and the full dataset, with attention to both shared experiences and divergent accounts. Throughout this process, themes were refined, reorganized, or redefined when necessary to improve conceptual coherence and explanatory depth. Analytic discussions among the research team were conducted throughout the process to facilitate reflexivity, explore alternative interpretations, and examine assumptions influencing the analysis. These discussions were not intended to achieve coding consensus or establish a single correct interpretation. Instead, they supported a deeper and more nuanced understanding of participants’ experiences. NVivo 15 was used to organize transcripts, codes, analytic memos, and thematic development.

To illustrate the analytic process, one example is provided. When reviewing participants’ descriptions of inpatient daily life, repeated accounts of “having nothing to do,” “repetitive daily routines,” and “waiting for time to pass” were initially coded as experiences of unstructured time, monotony, and reduced sense of purpose. These codes were subsequently examined across transcripts and interpreted as reflecting a broader pattern in which the present moment was experienced as stagnant and disconnected from meaningful engagement. Together with related accounts describing how activities and interpersonal interactions provided structure and meaning to daily life, these interpretations contributed to the development of the theme “Present experience between stagnation and relational engagement.”

This analytic process was iterative, involving repeated movement between individual extracts, developing codes, candidate themes, and the entire dataset. Themes were refined through reflexive discussions among the research team to explore alternative interpretations, examine assumptions, and strengthen the conceptual coherence of the analysis.

### 2.6 Rigor and trustworthiness

The quality of the qualitative analysis was supported through several reflexive practices. Prolonged engagement with the transcripts, repeated review of coded extracts, and maintenance of analytic memos supported familiarity with participants’ accounts and the development of themes. Attention was given not only to recurring patterns but also to divergent experiences that challenged emerging interpretations.

Reflexivity was considered throughout the research process. The research team reflected on how their clinical backgrounds, professional experiences, and assumptions regarding recovery and temporal change might influence data interpretation. The team recognized that its mental health nursing and recovery-oriented perspective might sensitize the analysis toward accounts of improvement and adaptation. Reflexive discussions therefore explicitly considered persistent distress, ambivalence, and accounts that did not fit an improvement-oriented interpretation. Team discussions were used to make these assumptions explicit, explore alternative readings of the data, and refine thematic interpretations rather than to establish coding agreement. An audit trail was maintained using NVivo 15, including transcripts, coding records, analytic notes, and documentation of theme development and revision. The final themes were grounded in participants’ narratives and supported by representative quotations.

## 3 Results

**Figure 1:**
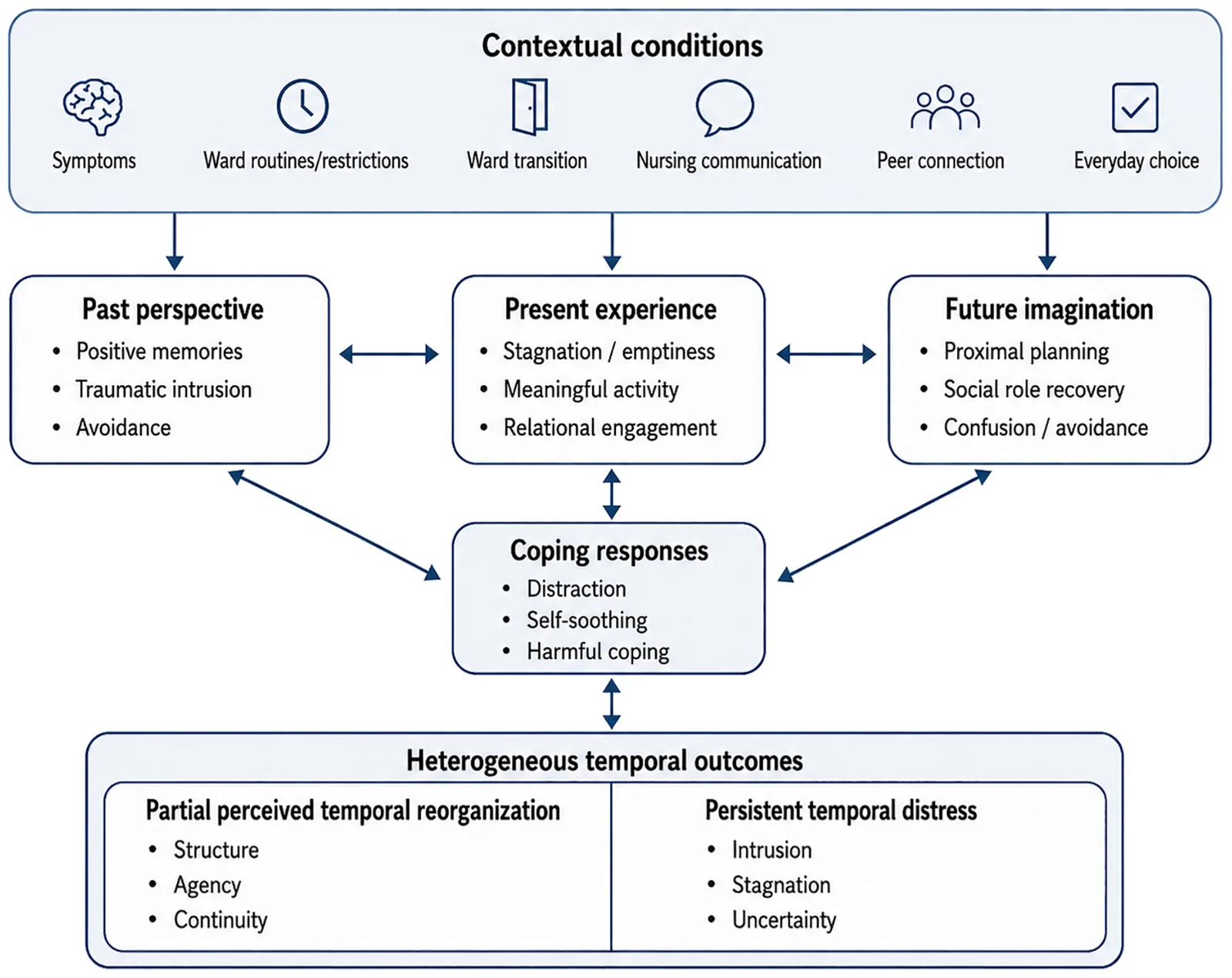
Non-linear model of perceived temporal reorganization during psychiatric hospitalization. The model illustrates the interrelated and heterogeneous nature of participants’ temporal experiences. Past, present, and future orientations interacted with coping responses and contextual conditions within the inpatient setting. Participants’ accounts indicated both partial perceived temporal reorganization and persistent temporal distress. The arrows represent interpretive relationships within participants’ narratives rather than causal pathways or universal stages of recovery.

Based on the thematic analysis, five themes were generated: the duality of the past perspective, the reshaping of present experience, the folding and extension of future images, coping strategies amid temporal distress, and perceived shifts in temporal organization. Together, these themes show that participants’ temporal experiences during hospitalization were heterogeneous and sometimes contradictory. Some participants described partial movement toward greater temporal structure, agency, or future imaginability, whereas others continued to experience traumatic intrusion, ward-related emptiness, future uncertainty, or harmful coping. Therefore, perceived temporal reorganization is presented here as a partial and non-linear experience rather than a universal trajectory of improvement. The conceptual models for topic thumbnails and time reorganization are shown in **Table 2** and **Figure 1** below.

**Table 2:** Theme overview.

Theme	subtheme	Representative quotation
The duality of the past perspective: emotional refuge and traumatic intrusion	Positive recollections as psychological support for illness management	“Good memories make me feel better and keep my emotions stable. They also give me more confidence to manage my illness and follow medical advice.” (P2)
	Involuntary intrusion of traumatic memories and erosion of the present	“When I think about my past, my whole body turns cold … Life feels meaningless, I do not want to do anything.” (P1)
	Avoiding the past as a defensive mechanism	“I do not want to think back on the past. It brought me too much pain.” (P4)
The reshaping of present experience: from perceived stagnation to relational awakening	Temporal stagnation and feelings of emptiness in a closed ward	“Whenever there is nothing to do, it feels really boring.” (P6)
	Therapeutic relationships and activities that light up time	“When I do rehabilitation training with nurses or talk with other patients about something meaningful, I feel happy, and time feels full.” (P11)
The folding and extension of future images: contraction under uncertainty and gradual reconstruction	Stabilize first, then extend: a narrowed future horizon	“First make life stable, and then slowly plan what comes next.” (P5)
	Reconstructing the future around recovery of social functioning	“I want to take care of my family and my children, and make myself stronger.” (P7)
	Confusion and avoidance regarding an unknown future	“I am still very confused about the future.” (P9)
Coping strategies amid temporal distress: passive endurance and active self-soothing	Passing time through attentional distraction	“I read, walk around, or just daydream, so that time can pass a little faster.” (P12)
	Self-soothing with introspective features	“I calm myself down first … and reflect once I have composed myself.” (P2)
	Harmful coping signaling deeper psychological crisis	“I pick at my hand … until my hand goes numb.” (P13)
Perceived shifts in temporal organization: emerging agency and changing self-understanding	From feeling controlled by time to experiencing greater everyday agency	“Now I can arrange my own time and make each day feel full.” (P4)
	Perceived turning points associated with symptom changes and ward transitions	“After I was transferred to the level-two ward, I suddenly felt much calmer.” (P8)
	Participants’ perceived changes in self-understanding and emotional response	“I have learned tolerance and understanding.” (P11)

### 3.1 Theme 1. The duality of the past perspective: emotional refuge and traumatic intrusion

Participants did not relate to the past in a uniformly negative or nostalgic manner; rather, they moved between drawing comfort from the past and remaining trapped in the past. The past could function as a reservoir of emotional stability, but it could also become an abyss that reactivated negative symptoms. Some participants intentionally preserved positive memories and used them as resources for emotional stabilization and confidence in treatment. Others were repeatedly disturbed by painful experiences, with past memories directly undermining same-day mood, motivation, and sleep. Still others tried to avoid the past as much as possible in order to reduce emotional fluctuation.

#### 3.1.1 Positive recollections as psychological support for illness management

Some participants preferred to recall positive past experiences and regarded these memories as important resources for maintaining emotional stability and strengthening treatment adherence. “I tend to think of good things more easily and not bad ones. Good memories make me feel better and keep my emotions stable. They also give me more confidence to manage my illness and follow medical advice.”(P2) “I usually find that focusing on the good things helps me stay positive and gives me plenty of motivation every day. Occasionally, I think about past sorrows, but the positive energy always outweighs the negative.”(P4).

#### 3.1.2 Involuntary intrusion of traumatic memories and erosion of the present

Other participants reported that experiences such as troubled marriages, family conflict, and the death of relatives or friends repeatedly entered their minds, leaving them feeling irritable, powerless, lonely, or even hopeless. “When I think about my past, my whole body turns cold. It affects my life and my mood. Life feels meaningless, I do not want to do anything, and I feel especially lonely, like I am completely falling apart. At night I am especially frightened when I try to sleep.”(P1) “Every time I think about my family not understanding me and the arguments we’ve had, I feel really down. I always want to retreat into my own space, but I can’t seem to stop those images of our arguments from popping into my head.”(P15) “A broken marriage, unruly children, and unpleasant memories from the past are affecting my life now; I’m prone to hallucinations and sometimes even get lost in them.”(P8).

#### 3.1.3 Avoiding the past as a defensive mechanism

When faced with unbearable memories, some participants chose emotional distancing in order to maintain psychological balance in the present. “I do not want to think back on the past. It brought me too much pain, so now I try not to think about it as much as possible.”(P4) “I told my family that no one should bring up past experiences; I don’t want to relive those upsetting stories.”(P3).

### 3.2 Theme 2. The reshaping of present experience: from perceived stagnation to relational awakening

During early hospitalization or in the absence of external stimulation, participants often experienced the present as slow, blank, and devoid of meaning, a state of temporal stagnation. However, this stagnation was not fixed. When patients connected with peers, participated in rehabilitation activities, or received support from nurses, time was re-endowed with meaning and became fuller and more substantial.

#### 3.2.1 Temporal stagnation and feelings of emptiness in a closed ward

Participants commonly reported boredom, confinement, and lack of purpose in the closed ward, with some parts of the day feeling especially difficult to endure. “When the doctors have not come for rounds, or in the afternoon, I feel very bored. I just walk around in the activity room. Whenever there is nothing to do, it feels really boring…”(P6) “It feels like I am dreaming now. One dream ends and then there is another. I often cannot control my thoughts, and when I am clear-headed, my mind feels empty, as if there is nothing there.”(P15).

#### 3.2.2 Therapeutic relationships and activities that light up time

When participants talked with peers, took part in activities, or received attention from nurses, previously stagnant time became fuller and more meaningful. “When I do rehabilitation training with the nurses or talk with other patients about something meaningful, I feel happy, and time feels full.”(P11) “The nurses take great care of us every day, asking us if anything is bothering us and leading us through psychological rehabilitation exercises and little games. With them by our side, time flies by.”(P5) “I made a good friend in the hospital ward. We both came from single-parent families, and we’d confide in each other when things were tough. She was especially understanding, and having her there brought a ray of light into my otherwise dreary hospital stay.”(P1).

### 3.3 Theme 3. The folding and extension of future images: contraction under uncertainty and gradual reconstruction

Participants’ images of the future were clearly differentiated. Some had begun to reconstruct the future around recovery, family, study, and work, often with the primary orientation of first stabilizing the near future. Others, however, described confusion, blankness, and avoidance; for them, the future remained uncertain and even frightening.

#### 3.3.1 Stabilize first, then extend: a narrowed future horizon

Most participants lacked long-term plans, and their temporal horizon was largely confined to the short period after discharge, ranging from several months to half a year. “What I can picture most clearly is the first six months after discharge - first make life stable, and then slowly plan what comes next.”(P5) “After you’re discharged, make sure to take good care of yourself first, keep your emotions in check, take your medication regularly, and maintain a normal routine. Don’t set overly strict expectations for yourself—just take it one step at a time.”(P10).

#### 3.3.2 Reconstructing the future around recovery of social functioning

Patients in recovery began to orient the future toward rebuilding family roles and personal growth. “First, I need to recover from my illness and manage myself by following medical advice. Then I want to take care of my family and my children, and make myself stronger and more independent.”(P7) “I need to manage my condition on my own, keep my emotions in check, and—while I’m recovering—do whatever I can to ease the financial burden on my family.”(P2).

#### 3.3.3 Confusion and avoidance regarding an unknown future

Some participants said that they had no clear plan for the future and even felt fear or avoidance toward it. “I am still very confused about the future. I feel empty inside, and I do not know what I am supposed to do.”(P9) “Sometimes I feel that I still do not quite dare to face the future.”(P3).

### 3.4 Theme 4. Coping strategies amid temporal distress: passive endurance and active self-soothing

When facing negative emotions or the pressure of time that seemed impossible to endure, participants developed a variety of coping strategies in an attempt to regain control over the present experience. These strategies were used both to make time pass faster and to relieve emotion, restore calm, and recover a sense of control.

#### 3.4.1 Passing time through attentional distraction

Using basic activities to accelerate the subjective passage of time was the most common approach. “I read, walk around, or just daydream, so that time can pass a little faster.”(P12) “Talking with other patients or listening to music I like works pretty well for me. During hospitalization it usually helps.”(P8).

#### 3.4.2 Self-soothing with introspective features

Some participants described more reflective forms of self-soothing. “I find a quiet place, take deep breaths, meditate, and talk to myself. Little by little I calm down.”(P1) “I tried to calm myself down first, step back from the situation, find a comfortable environment, and reflect on things once I had composed myself.”(P2).

#### 3.4.3 Harmful coping signaling deeper psychological crisis

One participant, rather than participants generally, described a harmful form of coping during emotional overload. “I tell myself it is okay, and then I pick at my hand. I keep doing it until my hand goes numb, and only then do I slowly let go.”(P13).

### 3.5 Theme 5. Perceived shifts in temporal organization: emerging agency and changing self-understanding

Some participants described changes in their relationship with time during hospitalization. For these participants, time gradually became more structured, manageable, or connected with daily activities. However, these shifts were not described by all participants and should not be understood as a uniform process of improvement. Perceived temporal reorganization coexisted with continuing uncertainty, emotional distress, and uneven recovery of future orientation.

#### 3.5.1 From feeling controlled by time to experiencing greater everyday agency

Many participants mentioned that time had felt extremely slow and chaotic when they were first admitted, but later they began to develop the ability to structure and arrange their days. “Before, it felt as though time was pushing me along. Now I can arrange my own time and make each day feel full.”(P4) “When I had just been admitted, time felt very slow. Now there is something to do every day. Looking back, it feels as though I have become a different person.”(P6).

#### 3.5.2 Perceived turning points associated with symptom changes and ward transitions

Participants described changes in their experience of time alongside several contextual changes, including perceived symptom relief and transitions to less restrictive ward environments. Some participants associated feeling calmer and having more manageable daily routines with these changes. “From the day I was transferred from the level-one ward(Ward for Acute Care Patients) to the level-two ward(Ward for patients whose condition has stabilized), I suddenly felt much calmer, and time no longer felt so hard to get through.”(P8) Another participant similarly described that symptom relief and increased interaction with others coincided with a more meaningful daily experience: “About one week after I was admitted, I began to feel much better. Talking with other patients and being cared for so carefully by the nurses made every day feel meaningful.”(P2).

These accounts suggested that perceived changes in time experience were closely connected with participants’ perceptions of symptom changes, ward environment, and interpersonal support.

#### 3.5.3 Participants’ perceived changes in self-understanding and emotional response

Changes in the sense of time were not only about time passing faster or slower; they were also accompanied by changes in emotion, cognition, and the relationship with the self. “I am more mature now than before. My emotions are more stable, and I can accept myself better. I can gradually come to terms with many things.”(P11) “As time has changed, I have learned to think from other people’s perspectives. I am less stubborn now, and I have learned tolerance and understanding. I am no longer so easily affected emotionally by what other people say.”

## 4 Discussion

Participants’ accounts suggested that temporal experience during hospitalization was dynamic but not uniformly progressive. Some participants described greater temporal structure, meaningful engagement, and emerging agency alongside perceived symptom relief, relational support, structured activities, and changes in ward environment. Others continued to experience intrusive memories, ward-related emptiness, uncertainty about the future, or harmful coping. These findings suggest that subjective time in schizophrenia should be understood as emotional, relational, and context-sensitive, rather than as either a fixed deficit or a simple pathway toward recovery.

This study identified a dual relationship with the past among hospitalized patients with schizophrenia, corresponding to both Past-Negative and Past-Positive orientations in Zimbardo’s time perspective framework (5). Some participants described traumatic memories and adverse life experiences that continued to influence their present emotions, while others drew upon positive memories as sources of emotional regulation and confidence. Consistent with previous quantitative findings, a more negative orientation toward the past has been associated with greater psychological distress in schizophrenia (21, 22). Clinically, these findings suggest that reminiscence-based approaches should be applied cautiously, as recalling the past may also activate distressing experiences. Instead, trauma-informed autobiographical memory work may help patients identify meaningful past experiences and personal strengths, potentially enhancing their sense of agency and coping confidence in the context of illness. This suggestion represents a potential clinical implication derived from participants’ narratives rather than an intervention evaluated in the present study.

Zimbardo’s theory suggests that individuals with a Present-Fatalistic orientation may perceive their lives as constrained by external forces and limited possibilities for change (23, 24). In this study, participants’ experiences of temporal stagnation appeared to be associated not only with illness-related difficulties but also with contextual features of inpatient life, including ward restrictions, medication effects, and reduced opportunities for autonomous activity. However, participants also described variations in how they experienced present time. For some, interactions with peers, rehabilitation activities, and supportive nursing communication were associated with a greater sense of engagement and meaning in daily life. These accounts suggest that present temporality in inpatient settings is not simply an individual psychological characteristic, but an experience shaped through interactions between personal conditions and the surrounding environment (2).

Most participants in this study did not formulate distant or grand future plans. Instead, they focused on stable living in the months after discharge, regular medication use, caring for family, and restoring basic social functioning. This finding echoes previous research showing constrained future imagination and weakened future orientation in schizophrenia, but it also suggests that such future contraction should not be interpreted simply as negativity or deficit (8, 25). During hospitalization, a focus on proximal goals may represent a more realistic and protective way of organizing time. From Zimbardo’s perspective, this may be understood not merely as cognitive impairment but as an adaptive psychological defense. Faced with relapse risk, damaged social roles, and long-term uncertainty, patients compress the future into a manageable and executable range, which may reflect active regulation under conditions of limited psychological resources (26). At the same time, some participants still described a blank or avoided future, indicating that the recovery of future orientation is uneven and that some patients need external support to gradually build a future that feels imaginable and attainable.

An important contribution of this study is the identification of perceived changes in temporal organization during hospitalization. However, these changes should not be interpreted as a universal developmental trajectory or a direct effect of hospitalization. Participants described diverse experiences: some perceived greater temporal structure, more meaningful engagement with daily life, and increased agency, whereas others continued to experience intrusive memories, uncertainty, or difficulties imagining the future. These findings suggest that perceived temporal reorganization represents an individualized and context-dependent process through which patients make sense of their experiences across past, present, and future. Unlike previous research that has primarily emphasized deficits in temporal processing among people with schizophrenia, the present study highlights subjective temporality as an experiential dimension that may involve both disruption and partial reorganization (12). Rather than indicating psychological growth or recovery itself, temporal reorganization describes changes in how individuals perceive continuity, meaning, and agency within their current life circumstances.

### 4.1 Theoretical contribution: perceived temporal reorganization as a process of subjective temporality

Existing research on time perspective in schizophrenia has mainly focused on temporal orientations as measurable dimensions, such as past-negative, present-fatalistic, or future-oriented tendencies, and has examined their associations with symptoms and functioning (3, 12). Building on this literature, the present study used time perspective as a sensitizing framework to explore how patients themselves experienced the relationship among past, present, and future during psychiatric hospitalization.

Our findings extend previous work by suggesting that subjective temporality in schizophrenia is not only characterized by imbalanced temporal orientations but may also involve dynamic changes in how temporal experiences are connected and interpreted. Participants described how distressing past experiences could shape present emotional states, how meaningful activities and interpersonal relationships could influence the experience of present time, and how changes in present stability could affect the ability to imagine the future. Based on these narratives, we use the term perceived temporal reorganization to describe a participant-reported and context-dependent process in which the organization of past, present, and future experiences may shift. This concept is not equivalent to recovery, adaptation, improved self-identity, or balanced time perspective. Recovery represents a broader multidimensional process, whereas temporal reorganization specifically concerns changes in the subjective organization of time (27). Similarly, balanced time perspective refers to an adaptive temporal orientation profile, while temporal reorganization describes a process through which individuals make sense of temporal experiences within a particular life context.

Therefore, the theoretical contribution of this study is not to propose temporal reorganization as a new clinical outcome, but to highlight subjective temporality as a dynamic and relational aspect of psychiatric recovery experiences. This perspective may complement existing symptom-focused and function-oriented approaches by drawing attention to how patients experience continuity, meaning, and agency across time.

### 4.2 Clinical implications for inpatient psychiatric care

This study has implications for inpatient psychiatric assessment and care. First, clinicians should attend not only to symptom severity but also to the temporal focus that currently dominates the patient’s experience. For some individuals, intrusive past memories and helplessness appeared to erode the present; for others, empty ward time and lack of agency intensified stagnation; and for others, the future remained vague, blank, or frightening. Temporal assessment may therefore help identify different recovery needs that are not fully captured by symptom ratings alone.

Second, the findings suggest that patients’ experiences of time within inpatient settings are closely related to their daily activities, interpersonal relationships, and perceived opportunities for agency. Structured daily activities, meaningful interpersonal communication, even when brief, opportunities for peer connection, and carefully individualized opportunities for greater everyday choice may help patients experience ward time as more connected and manageable. Participants associated greater everyday agency with contextual changes such as perceived symptom stabilization, transitions to less restrictive ward environments, and increased engagement in meaningful activities. This has implications not only for nursing practice but also for ward routines, multidisciplinary care, and service design.

Third, future-oriented support may be most effective when it starts with proximal, concrete goals rather than distant life plans. Helping patients imagine the first days or weeks after discharge, rebuild daily rhythm, and identify manageable next steps may support a more attainable and less threatening sense of the future. In this way, inpatient psychiatric care may provide opportunities to explore and support patients’ subjective experiences of temporal continuity within broader psychosocial recovery processes.

### 4.3 Strengths and limitations

This study has several strengths. First, it focused on time perspective among hospitalized patients with schizophrenia, a topic that has received relatively little qualitative attention, and used patient narratives to reveal the dynamic organization of past, present, and future. In doing so, it supplements previous evidence dominated by quantitative measurement and deficit-oriented models. Second, the study included clinically stable participants from four closed wards, which increased the diversity of experiences represented in the sample. Third, the analysis addressed not only temporal imbalance but also participants’ perceived changes and reorganizations in temporal experience.

This study also has limitations. First, all participants were recruited from a single psychiatric hospital and were in a clinically stable phase; therefore, the findings may not represent the experiences of patients in acute episodes or those receiving care in other regions or under different care models. Second, the sample size was small and purposive sampling was used, so the findings are better suited to theoretical and experiential transferability than to statistical generalization. Third, the study was based on one-time interviews and could not examine longitudinally whether changes in time perspective were sustained after discharge. Finally, although repeated team discussion was used to enhance analytic credibility, thematic analysis inevitably remains shaped by the interpretive perspective of the researchers. Because the findings were derived from one-time interviews, they reflect participants’ retrospective accounts of temporal change during hospitalization rather than prospectively observed trajectories.

## 5 Conclusions

Subjective time perspective among hospitalized patients with schizophrenia was described by participants as dynamic and context-sensitive rather than fixed. Participants’ narratives revealed both persistent temporal distress and experiences consistent with partial temporal reorganization, involving perceived changes in continuity, meaning, and agency. These experiences were embedded within interactions among illness-related challenges, ward environments, interpersonal relationships, daily activities, and individual coping strategies. Rather than representing a universal pathway of recovery, psychological growth, or treatment-related change, perceived temporal reorganization highlights how patients interpret and negotiate their experiences across past, present, and future during psychiatric hospitalization.

## Data Availability

The datasets are not publicly available due to privacy and ethical restrictions. Requests to access the datasets should be directed to the corresponding author.
